# Evaluate the physiological role of tetrapyrroles precursor on growth, yield and some biochemical composition of two cultivars of *Vicia faba* L.

**DOI:** 10.1186/s12870-025-06418-9

**Published:** 2025-04-15

**Authors:** Mona G. Dawood, Mohamed E. El-Awadi, Mervat Sh. Sadak, Mahmoud A. Khater, Yasser R. Abdel-Baky

**Affiliations:** Botany Department, Agriculture and Biological Institute, National Research Centre, 33 El Bohouth St P.O. 12622, Dokki, Giza, Egypt

**Keywords:** 5-aminolevulinic acid, Tetrapyrroles, Chlorophyll, Faba bean, Seed quality

## Abstract

It is well known that 5-aminolevulinic acid (5ALA) is a non-protein amino acid and essential for the formation of biosynthesis of tetrahydropyrroles. So, two field experiments were carried out in a private farm at Sharkia Governorate to study effect of foliar spraying with 5ALA (1, 3, and 6 mgL^−1^) on both quality and economic characters of two cultivars of *Vicia faba* L. (Giza 843 and Nubaria 1). Results indicated that plants belong to Nubaria 1 cv. are characterized by significant increases in all components of photosynthetic pigments, indole acetic acid, free amino acids, seed yield /fed and straw yield/fed over those of Giza 843 cv. under control treatments. Notably, yielded seeds of Giza 843 cv. are characterized by significant increases in total carbohydrate and protein content than those of Nubaria 1 cv. Whereas, yielded seeds of Nubaria 1 cv. are characterized by significant increases in total phenolic content and vicine. Moreover, 5ALA treatments significantly increased most of all values of vegetative growth parameters, photosynthetic pigments, indole acetic acid, proline and free amino acids as well as seed and straw yield/fed, total carbohydrate and protein, and phenolic contents accompanied by significant decreases in vicine content of two faba bean cultivars relative to corresponding controls. On the other hand, the increments in most of investigated parameters were in opposite direction with concentration of 5ALA.The least concentration of 5ALA (1mg/L) was the most significant treatment in both cultivars. Since it increased seed yield by 17.86% and 72.27% in Giza 843 cv. and Nubaria 1 cv. respectively relative to corresponding controls. Regarding anti-nutritional substance called vicine, 5ALA at 3mg/L caused significant decrease in vicine content of Giza 843 cv. relative to control. It could be concluded that faba bean plants belong to Nubaria 1 cv. effectively responded to 5ALA at 1mg/L more than faba bean plants belong to Giza 843 cv.

## Introduction

It is well known that 5-aminolevulinic acid (5ALA) is a non-protein amino acid and essential for the formation of biosynthesis of tetrahydropyrroles, which include vitamin B12, hemoglobin, and chlorophyll in living organisms. It can be synthesized in all plants, animals and microorganisms at very low concentrations [1–3]. Moreover, 5ALA is a natural and environmentally friendly substance, at a low level, and possesses a wide-range of applications and market-development potentials because it has no harmful effects on humans and animals, is easily degradable, and does not leave residue in the ecological environment [4, 5]. In addition to its role as a regulator of plant growth, 5ALA is also involved in many other biological processes in plants which involved in the regulation of plant growth and development. So, 5-ALA has a great application potential in agricultural [1, 2, 6, 7]. It regulates a wide range of physiological processes of plants, such as seed germination [8], primary root elongation [9], photosynthesis and guard cell movement [4, 10], pollen germination and tube growth [5], stomatal opening [5, 11] and efficiency of photosynthetic process [12]. El-Metwally et al. [13] stated that 5-aminolevulinic acid treatments have the potentiality to induce the synthesis of photosynthesis pigments, indole acetic acid, phenolics and free amino acids, as well as quantity and quality of the yielded peanut seed. Additionally, it increased plant tolerance to almost all types of abiotic stresses [14] thereby enhanced plant biomass accumulation and increasing crop yield [9, 15, 16]. Its beneficial role in increasing plant biomass, photosynthesis process and quality under normal growth conditions has been established on cucmber [15], peanut [13] and grapevine [17].

Several researches indicated that 5ALA reduced the harmful effects of abiotic stress on plants, by enhancing photosynthetic efficiency, nutrient absorption, water transport, and antioxidant enzyme activities [14, 15, 18, 19]; and significantly protected plasma membranes against free reactive oxygen radicals [7, 20, 21]. It is worthy to note that application of 5ALA at low concentrations promoted plant growth and development either under normal or stressful conditions [3]. Previously, Hotta et al. [22] demonstrated that 5ALA at low levels (0.06—0.60 mM) increased fixation of CO_2_ in light, and suppressed the release of CO_2_ in darkness. According to Tavallali et al. [23], exogenous application of 5ALA enhanced plant growth and nutritional quality and pharmaceutical properties of *P. oleracea* plants because of its growth regulatory effects. Whereas 5ALA application—at relatively higher concentrations (i.e., 5—40 mM)- has herbicidal properties and is harmful to plants [3, 22]. In this regard, low levels of 5ALA (0.5 or 1 mg/L) boosted the biomass and the content of different bioactive substances in oilseed rape; whereas high levels of 5ALA (5 or 10 mg/L) caused oxidative stress, which is reduced to the growth of oilseed rape [24].

Faba bean (*Vicia faba*) is a significant crop that is grown extensively in many regions of the world, particular in North Africa, on mixed rain-fed dry land or in agro-pastoral systems. Egypt was the world's biggest importer of faba beans because Egyptians consume a significant amount of faba beans (about 14 g per person per day) which provides roughly 3 g of protein [25]. Further, faba bean is one of the most significant legumes with high nutritive value for humans and livestock, because of its richness in important nutrients such as starch, proteins, fatty acids, dietary fibers, minerals and vitamins [26, 27]. In addition,faba bean contains bioactive compounds and potent natural antioxidants such as phenolics and flavonoids [28].

It was noted that very limited data are available on the effects of 5-aminolevulinic acid on legume plants. Moreover, up to date, most research on the effects of 5ALA has focused on the physiological level, and little is known about the changes at the molecular level. Regarding its benefits, 5-aminolevulinic acid possesses a wide range of applications and market development potentials as it has no toxic effects on humans, livestock, or the environment; besides, it is easily degradable and residue-free in the ecological environment [29]. Therefore, to realize the mechanism of action of 5ALA on the main legume, such as faba bean, a specified study has to be carried out to deal with physiological, biochemical and molecular changes due to 5ALA treatment at different levels. An attempt to cover this point, this investigation aimed to compare the response of two cultivars of faba bean named Giza 843 and Nubaria 1 to foliar application of 5-aminolevulinic acid at different concentrations (0, 1, 3, and 6 mg/L), regarding the changes in physiological, biochemical, and molecular levels.

## Material and methods

### Chemicals

All chemicals used in the experiments were of analytical grade. 5-aminolevulinic acid (BioReagent, powder, ≥ 98%), dimethylamino benzoic acid, anthrone, ninhydrin, Folins- Ciocalteu reagent were purchased from Sigma Chemical Company (St. Louis, MO, USA). Acetone, methanol, ethanol, sulphuric acid, glacial acetic acid, proline, glycine, sodium carbonate, were purchasedfrom Greiner Diagnostic Gmbh-Germany and Bio-Diagnostic Company, Egypt.

### Plant materials

Seeds of two cultivars of *Vicia faba* L. (Giza 843 and Nubaria 1) were acquired from the Agricultural Research Center in Giza, Egypt.

#### Experimental procedure and growth conditions

Throughout the winter seasons of 2021/2022 and 2022/2023, two field studies were conducted in a private farmland in Sharkia Governorate to evaluate the impact of foliar application with 5ALA on the economic and quality characteristics of two *Vicia faba* cultivars. The investigated soil's physical and chemical properties (Table 1) were carried out in accordance with Cottenie et al. [30].

**Table 1:** Characteristics of the soil in Sharkia governorate, Egypt

Physical characteristicsAQ					
Texture	Clay	Silt	Sand	EC(ds.m−1)	pH
	(%)				
Clay	50.2	38.3	11.6	0.46	7.49
Chemical characteristics					
Cations (meq .1–1)			Anions (meq .1–1)		
Ca	Mg	Na	SO4	CL	HCO3
2.19	0.68	1.15	3.36	1.18	1.40
Macronutrient			Micronutrient (mg.kg−1)		
N (meq .1–1)	P (ppm)	K (ppm)	Zn	Fe	Mn
47.2	26.5	386	1.16	5.9	0.30

In regard to fertilization, during seed bed preparation, calcium superphosphate as P_2_O_5_ (15.5%) and potassium sulphate as K_2_O (48–52%) were incorporated at levels of 31 and 24 kg/fed, respectively, while ammonium nitrate (33.5%), a form of nitrogen fertilizer, was incorporated at a rate of 75 kg N/fed. During the two growing seasons, faba bean seeds were sown in November in hills 20 cm apart on both sides of the ridge. Fifteen days after seeding, the plants were thinned to ensure each hill had two plants. Six replicates of each treatment were used in the split plot design of the trials. The two cultivars were distributed at random in the main plot, and the 5ALA concentrations were assigned randomly in the sub plot. Each experimental unit was 10.5 m^2^as 3 m long and 3.5 m wide, and 60 cm apart between ridges. All the cultural procedures like main field preparation, nursery raising, irrigation; fertilization, plant protection, weeding etc. were carried out as recommended. Further, during the two experimental seasons, foliar application with three concentrations of 5ALA (1, 3, and 6 mgL^−1^) were applied twice at 45 and 60 days after sowing date.

### Collected data

During vegetative growth parameters, after 75 days of planting, plant samples were taken for the purpose of determining certain morphological features (plant height (cm), number of leaves and branches/plant, and dry weight of plant (g)). After the plant sample was dried for 48 h at 50 degrees Celsius, the dry weight was determined. Fresh leaves were gathered for determination photosynthetic pigments, and indole acetic acid (IAA). Whereas, total carbohydrate, total soluble carbohydrate, free amino acids, and proline content were determined in dry leaf tissues.

At harvest time, ten plants from each treatment were gathered to determine the number of pods/plant, number of seeds /pod, and weight of seeds and straw (Ton/fed). The yielded healthy dry seeds were ground and used to measure the contents of total carbohydrate, protein, phenolic compound, and vicine content.

### Biochemical analysis

#### Determination of photosynthetic pigments

The photosynthetic pigments content in terms of chlorophyll a and b and carotenoids were measured according Li and Chen [31]. The fresh leaf tissues were grounded and pestles by 80% acetone and the developing colour was measured using Spekol spectrocolourimeter VEB Carl Zeissat 470,646 and 665 nm.

#### Determination of indole acetic acid content

A known weight of the fresh leaf tissues was extracted with 85% cold methanol (v/v) for three times at 0 °C. The combined extracts were collected and made up to a known volume with cold methanol. Then add 1 ml of the methanolic extract to 4 ml of PDAB reagent (1 g of para-dimethylamino benzoic acid dissolve in 50 ml HCl, 50 ml of ethanol 95%) and left for 60 min in 30–40^0^C. The developing colour was measured using Spekol spectrocolourimeter VEB Carl Zeiss at 530 nm [32].

#### Determination of total carbohydrate

Determination of total carbohydrates was carried out according to Albalasmeh et al. [33]. A known mass (0.2- 0.5 g) of dried tissue was placed in a test tube, and then 10 ml of sulphuric acid (1N) was added. The tube was sealed and placed overnight in an oven at 100ºC. The solution was then filtered into a measuring flask (100 ml) and completed to the mark with distilled water. 1 ml of solution was transferred into test tube and treated with 1 ml of 5% aqueous phenol solution followed by 5 ml of concentrated sulphuric acid. The tubes were thoroughly shaken for ten minutes then placed in a water bath at 23–30ºC for 20 min. The optical density of the developed color was measured at 490 nm using Spekol spectrocolourimeter VEB Carl Zeiss.

#### Determination of total soluble sugars

Total soluble carbohydrate was extracted by overnight submersion of dry leaf tissue in 10 ml of 80% (v/v) ethanol at 25°C with periodic shaking, and centrifuged at 600g. The supernatant was evaporated till completely dried then dissolved in a known volume of distilled water to be ready for determination of soluble carbohydrates. 0.1 ml of ethanolic extract with 3.0 ml freshly prepared anthrone (150 mg anthrone + 100 ml 72% H_2_SO_4_) in boiling water bath for ten minutes and reading the cooled samples at 625 nm using Spekol spectrocolourimeter VEB Carl Zeiss [34]. Polysaccharides were calculated by the difference between total carbohydrate and total soluble carbohydrate.

#### Determination of total free amino acids

The total free amino acids were determined according to the method described by Tamayo and Bonjoch [35]. A known weight of dry leaf tissues was homogenized in ethanol (80%), then boiling for 10 min and centrifuged at 2000 × g for 10 min. Then 0.05 ml of the collected supernatant, add to 2 ml of ninhydrin reagent and boiled in water bath (15 min). The mixture was cooled at room temperature for cooling and made up to 10 ml using distilled water. The color developed read at 570 nm using glycine as a standard using using Spekol spectrocolourimeter VEB Carl Zeiss.

#### Determination of proline

Proline was assayed according to the method described by Kalsoom et al. [36]. 2ml of proline extract, 2ml of acid ninhydrin and 2ml of glacial acetic acid were mixed and incubated for 1 h in a boiling water bath followed by an ice bath. The absorbance was measured at 520 nm using Spekol spectrocolourimeter VEB Carl Zeiss. A standard curve was obtained using a known concentration of authentic proline.

#### Determination of protein

Total nitrogen was determined using the micro-Kjeldahl method, as described by AOAC [37]. Total protein content was calculated by multiplying nitrogen percent by 6.25.

#### Determination of phenolic contents

A known weight of dry powdered seeds was extracted using 85% cold methanol (50 ml v/v) for three times at 90°C. The combined extracts were collected and made-up to a known volume with cold methanol. Then one ml of the extract was mixed with 0.5 ml Folins Ciocalteu reagent, shake, and allowed to stand for 3 min. Then 2 ml of saturated sodium carbonate (Na_2_CO_3_) was added to each tube followed by distilled water, shaken and left for 60 min. The absorbance was determined at 750 nm using using Spekol spectrocolourimeter VEB Carl Zeiss as described by Gonza'lez et al. [38].

#### Determination of vicine content

One gram of dried seeds was suspended in 30ml of 4% (w/v) phosphoric acid, chopped in a Warring blender, homogenized and centrifuged. The protein-free extract was diluted in 0.1 N HC1 and the optical density was measured using Spekol spectrocolourimeter VEB Carl Zeiss at 273.5 nm against 0.1 N HC1 as mentioned by Collier [39].

$$\mathrm{mg}\;\mathrm{total}\;\mathrm{vicine}/\mathrm g\;\mathrm{sample}\:=\:\mathrm O.\mathrm D.\:\times\:322\;/13.6\:\times\:103,\;\mathrm{where}\;322\:=\:\mathrm{molecular}\;\mathrm{weight}\;\mathrm{of}\;\mathrm{vicine};\;13.6\:\times\:103\:=\:\mathrm{molar}\;\mathrm{absorption}\;\mathrm{of}\;\mathrm{pure}\;\mathrm{vicine}.$$

### Genetical studies

#### ISSR amplification and PCR procedures

Fresh and young leaves of faba bean were used to isolate genomic DNA using plant genomic DNA extraction kit according to Dawood et al. [40] and Sadak et al. [41]. A total of 10 ISSR primers were screened for the production of polymorphic products from all treated plants under study, but 5 ISSR primers ((CT)_8_ GC, (CA)_6_AC, (GA)_6_CC, (GT)_6_CC and (CAC)_3_ GC) showed positive results so that they used in this study. However, the primers were chosen upon the production of distinct and reproducible bands in PCR reactions.

PCR procedures were done and programmed as described by Abdalla et al. [42], Dawood et al. [40] and Sadak et al. [41]. However, ISSR amplification reactions were carried out in15µl volume containing 1 µl DNA (40ng), 7.5µl MasterMix (Gene Direxone PCRTM), 1 µl template DNA and 1 µl primer. The amplification reaction consisted of aninitial denaturation step at 94°C for 7min,followed by 35cycles of 30s. at 94°C (denaturation), 45s. at 52°C (annealing) and 2min at 72°C (extension) followed by a final extension step at 72°C for 5min. Amplification products were electrophoresed on1.5% agarose in 1 × TBE buffer. The gels were stained with ethidium bromide and documented using gel documentation system. Each experiment was repeated twice with each primer and those primers which gave reproducible finger prints were considered for data analysis.

#### Polymorphism percentage was calculated according to this equation

$$\mathrm{PB}\%\:=\:(\mathrm{UB}\:+\:\mathrm{PB})/\mathrm{Total}\;\mathrm{bands}$$

Where PB% = polymorphism percentage, UB = number of unique bands, PB = number of polymorphic bands

### Statistical analysis

The statistical analysis of average data from two seasons using a spilt plot design was done according to Snedecor and Cochran [43]. Standard techniques were employed to perform the analysis of variance and the differences between the means were assessed using the Duncan multiple range test at a significance level of *P* = 0.05, utilizing the MSTATC-C [44] program.

## Results

It was noted from Table 2 that no significant differences in values of all vegetative growth parameters of two faba bean cultivars except number of leaves/plant. Moreover, 5ALA at 1 and 3mg/L significantly increased all investigated vegetative growth parameters relative to control. Whereas, 5ALA at 6 mg/L caused significant increases in plant height and number of leaves/plant and non significant increases in branches number/plant and plant dry weight.

**Table 2:** Impact of different concentrations of 5-aminolevulinic acid on some vegetative growth parameters of two faba bean cultivars

Treatments		Plant height (Cm)	Number of branches/plant	Number of Leaves/plant	Plant dry weight (g)
**Effect of cultivars**					
**Giza 843**		**44.83 ± 2.64 a**	**2.92 ± 0.70 a**	**96.83 ± 2.61 a**	**18.23 ± 2.24 a**
**Nubaria 1**		**42.41 ± 1.80 a**	**2.83 ± 0.76 a**	**82.08 ± 1.84 b**	**18.31 ± 2.30 a**
**Effect of 5- aminolevulenic acid**					
**5- aminolevulenic acid (mg/L)**	**0**	**36.33 ± 1.41c**	**2.33 ± 0.45 b**	**62.33 ± 2.51 d**	**16.26 ± 0.93 c**
	**1**	**50.17 ± 1.63 a**	**3.67 ± 0.45 a**	**104.0 ± 1.70 a**	**21.34 ± 0.54 a**
	**3**	**45.33 ± 0.93 b**	**3.17 ± 0.21 a**	**97.83 ± 0.95 b**	**18.65 ± 0.52 b**
	**6**	**42.67 ± 1.64 b**	**2.33 ± 0.49 b**	**93.67 ± 2.17c**	**16.72 ± 0.49 c**
**Effect of interaction between cultivars and 5- aminolevulenic acid**					
**Giza 843**					
**5- aminolevulenic acid (mg/L)**	**0**	**38.67 ± 1.15 d**	**2.330.58 b**	**62.00 ± 2.65 e**	**16.53 ± 1.15 dc**
	**1**	**52.00 ± 2.00 a**	**3.67 ± 0.58 a**	**114.7 ± 3.06 a**	**21.30 ± 0.35 a**
	**3**	**45.33 ± 1.15 bc**	**3.33 ± 0.26 a**	**107.3 ± 2.08 b**	**18.69 ± 0.69 b**
	**6**	**43.33 ± 1.53 c**	**2.33 ± 0.58 b**	**103.3 ± 1.53 b**	**16.40 ± 0.46 dc**
**Nubaria 1**					
**5- aminolevulenic acid (mg/L)**	**0**	**34.00 ± 1.73 e**	**2.33 ± 0.58 b**	**62.67 ± 3.06 e**	**16.00 ± 0.46 d**
	**1**	**48.33 ± 2.08 ab**	**3.67 ± 0.58 a**	**93.33 ± 2.08 c**	**21.56 ± 0.64 a**
	**3**	**45.33 ± 2.08 bc**	**3.00 ± 0.58 ab**	**88.33 ± 1.15 d**	**18.62 ± 0.23 b**
	**6**	**42.00 ± 2.00 cd**	**2.33 ± 0.58 b**	**84.00 ± 2.65 d**	**17.04 ± 0.05 c**

Means and Standard division followed by the same letter for each tested parameter are not significantly different by Duncan multiple range test at *P* = *0.05*

Regarding interaction between faba bean cultivars and different concentrations of 5ALA, it was noted that 1 and 3 mg/L 5ALA significantly increased all values of vegetative growth parameters of two faba bean cultivars relative to corresponding controls. The least concentration of 5ALA (1mg/L) was the most significant treatment in both cultivars. Since, it increased plant dry weight of Giza 843 cv. from 16.53 to 21.30g by 28.85% and that of Nubaria 1 cv. from 16.00 to 21.56 g by 34.75% relative to corresponding controls. These results indicate that response of the growth of Nubaria 1 cv. to 5ALA treatments (1mg/L) was more pronounced than that of Giza 846 cv. The increments in all vegetative growth parameters under investigation are in opposite direction with concentration of 5ALA. Where, the least increases in all vegetative growth parameters under investigation were recorded due to 5ALA at 6mg/L.

It was noted from Table 3 that there are significant differences in all components of photosynthetic pigments between the two faba bean cultivars. Specifically, the Nubaria1 cv. exhibited notable increases in all photosynthetic pigment components compared to the Giza 843 cv. Notably, 5ALA at all concentration caused significant increases in all components of photosynthetic pigments. Where, effect of 5ALA at 1mg/L was > 5ALA at 3mg/L > 5ALA at 6mg/L.

**Table 3:** Impact of different concentrations of 5-aminolevulinic acid on photosynthetic pigments of two faba bean cultivars

Treatments		Chlorophyll A	Chlorophyll B	Carotenoids	Total photosynthetic pigments
**mg/g fresh leaf tissues**					
**Effect of cultivars**					
**Giza 843**		**1.79 ± 0.38 b**	**0.58 ± 0.11 b**	**0.72 ± 0.16 b**	**3.10 ± 0.64 b**
**Nubaria 1**		**1.98 ± 0.36 a**	**0.69 ± 0.14 a**	**0.80 ± 0.19 a**	**3.48 ± 0.69a**
**Effect of 5- aminolevulenic acid**					
**5- aminolevulenic acid (mg/L)**	**0**	**1.42 ± 0.06 c**	**0.47 ± 0.001b**	**0.56 ± 0.01 c**	**2.46 ± 0.25 c**
	**1**	**2.26 ± 0.37 a**	**0.77 ± 0.05 a**	**0.92 ± 0.14 a**	**3.96 ± 0.62 a**
	**3**	**2.13 ± 0.05 a**	**0.72 ± 0.01 a**	**0.87 ± 0.02 a**	**3.72 ± 0.28 a**
	**6**	**1.74 ± 0.05 b**	**0.56 ± 0.01 ab**	**0.70 ± 0.02 b**	**3.01 ± 0.79 b**
**Effect of interaction between cultivars and 5- aminolevulenic acid**					
**Giza 843**					
**5- aminolevulenic acid (mg/L)**	**0**	**1.37 ± 0.08 e**	**0.44 ± 0.02 b**	**0.53 ± 0.04 e**	**2.34 ± 0.14 f**
	**1**	**2.14 ± 0.64 ab**	**0.69 ± 0.08 ab**	**0.85 ± 0.24 abc**	**3.69 ± 0.97 abc**
	**3**	**2.00 ± 0.11 abc**	**0.67 ± 0.01 ab**	**0.82 ± 0.02 bc**	**3.50 ± 0.13 bcd**
	**6**	**1.67 ± 0.09 cde**	**0.53 ± 0.02 ab**	**0.67 ± 0.03 de**	**2.88 ± 0.14 def**
**Nubaria 1**					
**5- aminolevulenic acid (mg/L)**	**0**	**1.48 ± 0.24 de**	**0.52 ± 0.07ab**	**0.58 ± 0.09 e**	**2.58 ± 0.4 ef**
	**1**	**2.39 ± 0.08 a**	**0.86 ± 0.60 a**	**0.97 ± 0.02 a**	**4.23 ± 0.004 a**
	**3**	**2.25 ± 0.29 a**	**0.78 ± 0.01 ab**	**0.91 ± 0.07 ab**	**3.95 ± 0.36 ab**
	**6**	**1.80 ± 0.12 bcd**	**0.60 ± 0.04 ab**	**0.73 ± 0.05 cd**	**3.13 ± 0.22 cde**

Means and Standard division followed by the same letter for each tested parameter are not significantly different by Duncan multiple range test at *P* = *0.05*

Regarding interaction between faba bean cultivars and different concentrations of 5ALA, it was noted that 5ALA treatments at all concentrations caused marked increased all values of photosynthetic pigments of two faba bean cultivars relative to corresponding controls. Since, 5ALA at 1mg/L significantly increased total photosynthetic pigments of Giza 843 cv. from 2.34 to 3.69 mg/g by 57.69% and of Nubaria 1cv. from 2.58 to 4.23 mg/g by 63.95% relative to corresponding controls. These results indicate that response of photosynthetic pigments of Nubaria 1 cv. to 5ALA treatments was more pronounced than that of Giza 846 cv. Meanwhile, 5ALA at 6mg/L caused non significant increases in total photosynthetic pigments of two faba bean cultivars relative to corresponding controls.

Table 4 shows that leaf tissues of Nubaria 1 cv. was characterized by higher significant values in indole acetic acid and free amino acids over those of Giza 843 cv. Whereas, Giza 843 cv. showed significant increase in proline content than that of Nubaria 1 cv. Non significant difference in total carbohydrate appeared between two cultivars. Moreover, all applied treatments significantly increased IAA, total free amino acids, proline, total carbohydrate, soluble carbohydrate and polysaccharides. 5ALA treatment at 1mg/L is the most optimum treatments in increasing IAA and total carbohydrates.

**Table 4:** Impact of different concentrations of 5-aminolevulinic acid on some biochemical composition of leaf tissues of two faba bean cultivars

Treatments		IAA	Free amino acids	Proline	Total carbohydrate	Total soluble carbohydrate	Polysaccharide
		**μg/100 g**	**mg/100 g**		**%**		
**Effect of cultivars**							
**Giza 843**		**40.47 ± 5.83b**	**259.7 ± 25.81 b**	**41.85 ± 7.78 a**	**23.72 ± 3.06 a**	**3.39 ± 0.77 b**	**20.33 ± 1.86 a**
**Nubaria 1**		**47.98 ± 7.62a**	**285.9 ± 7.62 a**	**40.20 ± 10.57b**	**23.40 ± 1.94 a**	**4.78 ± 0.96 a**	**19.12 ± 1.04 a**
**Effect of 5- aminolevulenic acid**							
**5- aminolevulenic acid (mg/L)**	**0**	**33.62 ± 041 d**	**246.3 ± 097 d**	**27.37 ± 0.57 d**	**20.35 ± 0.20 c**	**2.72 ± 0.004 c**	**17.63 ± 0.21 b**
	**1**	**52.55 ± 0.56a**	**282.4 ± 0.61 b**	**45.65 ± 0.34 b**	**26.18 ± 0.04 a**	**4.83 ± 0.03 a**	**20.91 ± 0.06 a**
	**3**	**47.59 ± 0.66b**	**272.2 ± 0.33 c**	**39.21 ± 0.09 c**	**23.01 ± 0.06 b**	**4.14 ± 0.02 b**	**19.30 ± 0.08 ab**
	**6**	**43.13 ± 0.33 c**	**290.2 ± 1.00 a**	**51.88 ± 0.34 a**	**25.88 ± 3.45 a**	**4.66 ± 0.09 b**	**21.05 ± 1.09 a**
**Effect of interaction between cultivars and 5- aminolevulenic acid**							
**Giza 843**							
**5- aminolevulenic acid (mg/L)**	**0**	**32.01 ± 0.50 g**	**217.8 ± 1.18 f**	**29.34 ± 0.70 g**	**19.89 ± 0.25 d**	**2.260 ± 0.01 f**	**17.63 ± 0.26 c**
	**1**	**47.76 ± 0.81 c**	**276.4 ± 0.66 d**	**42.26 ± 0.42 d**	**26.18 ± 4.22 a**	**3.95 ± 0.11 d**	**22.23 ± 1.33 a**
	**3**	**43.25 ± 0.81d**	**259.9 ± 0.75 e**	**38.26 ± 0.42 f**	**23.01 ± 0.04 bc**	**3.12 ± 0.03 e**	**19.89 ± 0.08abc**
	**6**	**38.85 ± 0.40e**	**284.8 ± 0..40 c**	**50.94 ± 0.10 b**	**25.80 ± 0.08 ab**	**4.24 ± 0.02 c**	**21.56 ± 0.10 ab**
**Nubaria 1**							
**5- aminolevulenic acid (mg/L)**	**0**	**35.23 ± 0.39 f**	**274.9 ± 0.50 d**	**25.39 ± 0.55 h**	**20.81 ± 0.03 cd**	**3.18 ± 0.08 e**	**17.63 ± 0.11 c**
	**1**	**57.35 ± 0.50a**	**288.5 ± 0.85 b**	**40.15 ± 0.15 e**	**25.04 ± 0.02 ab**	**5.70 ± 0.01 a**	**19.87 ± 0.16abc**
	**3**	**51.93 ± 0.69b**	**284.5 ± 2.58 c**	**49.05 ± 0.10 c**	**23.78 ± 0.08 ab**	**5.17 ± 0.16 b**	**18.70 ± 0.14 bc**
	**6**	**47.40 ± 1.25 c**	**295.6 ± 0.76 a**	**52.83 ± 0.65 a**	**25.96 ± 0.09 a**	**5.07 ± 0.06 b**	**20.26 ± 0.08abc**

Means and Standard division followed by the same letter for each tested parameter are not significantly different by Duncan multiple range test at *P* = *0.05*

Regarding interaction between faba bean cultivars and different concentrations of 5ALA, it was noted that application of 5ALA at all concentrations significantly increased indole acetic acid, proline and free amino acids in both cultivars of faba bean relative to corresponding controls. 5ALA at 1mg/L was the most pronounced treatments, since; it increased indole acetic acid from 32.01 to 47.67 μg/100g by 49.12% in Giza 843cv. and in Nubaria 1 cv. from 35.23 to 57.35 μg/100g by 62.78% relative to corresponding controls. Clearly, response of IAA of Nubaria 1 cv. to 5ALA treatments was more effective than response of Giza 843 cv. In addition, all applied treatments significantly increased total carbohydrate, and total soluble carbohydrate of two faba bean cultivars. The highest significant increases in total carbohydrate were attained by 1 mg/L 5ALA in Giza 843 cv. and by 3mg/ L 5ALA in Nubaria 1 cv. since, 1 mg/L 5ALA significantly increased total carbohydrate in Giza 843 cv. from 19.89 to 26.18% by 31.26% and 3mg/ L 5ALA significantly increased total carbohydrate in Nubaria 1 cv. from 20.81 to 25.96% by 24.74%.

It was noted from Table 5 that Nubaria 1 cv. was characterized by higher significant value of seed and straw yield/fed than those of Giza 843 cv. All applied treatments significantly increased values of seed and straw yield/fed, and these values are in opposite direction with concentrations of 5ALA.

**Table 5:** Impact of different concentrations of 5-aminolevulinic acid on seed yield and yield components of two faba bean cultivars

Treatments		Number of pods/plant	Number of seeds/pod	Seed yield (Tons/fed)	Straw yield (Tons/fed)
**Effect of cultivars**					
**Giza 843**		**12.58 ± 2.22 a**	**3.333 ± 0.66 a**	**1.21 ± 0.09 b**	**1.86 ± 0.40 b**
**Nubaria 1**		**11.42 ± 1.75 a**	**4.083 ± 0.64 a**	**1.37 ± 0.27 a**	**2.27 ± 0.55 a**
**Effect of 5-aminolevulenic acid**					
**5- aminolevulenic acid (mg/L)**	**0**	**12.17 ± 0.95 b**	**3.833 ± 0.46 a**	**1.05 ± 0.33 d**	**1.47 ± 0.16 d**
	**1**	**14.17 ± 0.47 a**	**3.667 ± 0.41 ab**	**1.52 ± 0.19 a**	**2.63 ± 0.29 a**
	**3**	**12.00 ± 1.29 b**	**3.167 ± 0.42 b**	**1.36 ± 0.12 b**	**2.33 ± 0.32 b**
	**6**	**9.667 ± 0.92 c**	**4.167 ± 0.73 a**	**1.23 ± 0.06 c**	**1.84 ± 0.32 c**
**Effect of interaction between cultivars and 5- aminolevulenic acid**					
**Giza 843**					
**5- aminolevulenic acid (mg/L)**	**0**	**12.67 ± 1.15 abc**	**3.33 ± 0.58 bc**	**1.10 ± 0.07 de**	**1.46 ± 0.20 d**
	**1**	**14.67 ± 0.58 a**	**3.00 ± 0.50 c**	**1.29 ± 0.10 c**	**2.38 ± 0.17 bc**
	**3**	**13.67 ± 1.53 ab**	**3.00 ± 0.50 c**	**1.24 ± 0.04 c**	**2.04 ± 0.10 c**
	**6**	**9.33 ± 1.15 d**	**4.00 ± 0.87 a**	**1.20 ± 0.07 cd**	**1.56 ± 0.20 d**
**Nubaria 1**					
**5- aminolevulenic acid (mg/L)**	**0**	**11.67 ± 1.53 bcd**	**4.33 ± 0.58 a**	**1.01 ± 0.07 e**	**1.49 ± 0.20 d**
	**1**	**13.67 ± 0.58ab**	**4.33 ± 0.58 a**	**1.74 ± 0.06 a**	**2.88 ± 0.17 a**
	**3**	**10.33 ± 1.53 cd**	**3.33 ± 0.58 b**	**1.48 ± 0.06 b**	**2.62 ± 0.20 ab**
	**5**	**10.00 ± 1.00 d**	**4.33 ± 0.58 a**	**1.27 ± 0.04 c**	**2.12 ± 0.17 c**

Means and Standard division followed by the same letter for each tested parameter are not significantly different by Duncan multiple range test at *P* = *0.05*

Regarding interaction between faba bean cultivars and different concentrations of 5ALA, it was noted that application of 5ALA at all concentrations significantly increased seed yield /fed and straw yield/fed of two faba bean cultivars relative to corresponding controls. The optimum treatment was 5ALA at 1mg/L, since; it significantly increased seed yield of Giza 843 cv. by 17.86% and seed yield /fed of Nubaria 1 cv by 72.27% relative to corresponding controls. It is clear, response of Nubaria 1 cv. to low concentration of 5ALA was more pronounced than the response of Giza 843 cv., that reflected in increasing the amount of seed yield /fed.

It was noted from Table 6 that yielded seeds of Giza 846 cv. was characterized by significant increases in total carbohydrate and protein content than those of Nubaria 1 cv. whereas, yielded seeds of Nubaria 1 cv. yielded seeds of was characterized by significant increases in total phenolic content and vicine. All applied treatments significantly increased total carbohydrate, protein content and total phenolic content accompanied by significant decrease in anti-nutritional compound (vicine). The most optimum treatment was 1 mg/l 5ALA.

**Table 6:** Impact of different concentrations of 5-aminolevulinic acid on some biochemical composition of the yielded seeds of two faba bean cultivars

Treatments		Total carbohydrate (%)	Protein content (%)	Phenolic content (mg/g)	Vicine content (mg/100 g)
**Effect of cultivars**					
**Giza 843**		**49.77 ± 1.65 a**	**23.35 ± 1.50 a**	**61.33 ± 1.31 b**	**313.90 ± 1.35b**
**Nubaria 1**		**47.55 ± 2.45 b**	**22.61 ± 1.60 b**	**68.35 ± 2.70 a**	**438.00 ± 1.85a**
**Effect of 5- aminolevulenic acid**					
**5- aminolevulenic acid (mg/L)**	**0**	**46.05 ± 0.41 d**	**20.88 ± 0.42 d**	**50.97 ± 0.19 d**	**398.80 ± 2.21 a**
	**1**	**51.02 ± 0.44 a**	**24.88 ± 0.23 a**	**61.30 ± 0.08 c**	**371.40 ± 1.29 b**
	**3**	**50.15 ± 0.53 b**	**23.81 ± 0.08 b**	**74.76 ± 0.53 a**	**357.60 ± 1.03 b**
	**6**	**49.76 ± 0.78 c**	**22.36 ± 0.19 c**	**72.34 ± 0.78 b**	**376.10 ± 0.47 b**
**Effect of interaction between cultivars and 5- aminolevulenic acid**					
**Giza 843**					
**5- aminolevulenic acid (mg/L)**	**0**	**47.35 ± 0.05 c**	**21.00 ± 0.52 e**	**53.38 ± 0.23 e**	**328.40 ± 2.70 c**
	**1**	**51.85 ± 0.10 a**	**25.05 ± 0.10 a**	**59.65 ± 0.10 d**	**309.30 ± 1.59 cd**
	**3**	**50.10 ± 0.25 b**	**24.42 ± 0.23 b**	**69.00 ± 0.65 b**	**294.10 ± 1.26 d**
	**6**	**49.76 ± 0.08 b**	**22.96 ± 0.28 c**	**63.30 ± 0.95 c**	**324.10 ± 0.57 cd**
**Nubaria 1**					
**5- aminolevulenic acid (mg/L)**	**0**	**44.74 ± 0.10 e**	**20.76 ± 0.09 e**	**48.55 ± 0.20 f**	**469.20 ± 0.50 a**
	**1**	**50.19 ± 0.05 b**	**24.70 ± 0.23 ab**	**62.94 ± 0.41 c**	**433.60 ± 2.05 b**
	**3**	**49.76 ± 0.08 b**	**23.21 ± 0.10 c**	**80.51 ± 0.83 a**	**421.20 ± 0.34 b**
	**6**	**45.50 ± 0.35 d**	**21.76 ± 0.08 d**	**81.39 ± 0.96 a**	**428.10 ± 1.26 b**

Means and Standard division followed by the same letter for each tested parameter are not significantly different by Duncan multiple range test at *P* = *0.05*

Regarding interaction between faba bean cultivars and different concentrations of 5ALA, it was noted that application of 5ALA at all concentrations significantly increased total carbohydrate and protein content of both cultivars relative to corresponding controls. It was noted that1 mg/l 5ALA was the most pronounced treatments that caused the highest significant increases in total carbohydrate in both faba bean cultivars relative to corresponding controls.

It is clear from Table 6 that all applied treatments significantly increased phenolic content of both faba bean cultivars. Regarding anti-nutritional substance called vicine, Table 6 showed that Nubaria 1 cv was characterized by higher significant value of vicine than that of Giza 843 cv. under control treatments. Whereas, 5ALA at 3 mg /L caused significant decrease in vicine content of Giza 843 cv. relative to control whereas, 1 mg/ L and 6mg/ L5ALA caused non significant decrease. On the other hand, all applied treatments caused significant decreases in vicine content of Nubaria 1 cv. relative to control specially at 3mg/L.

**Fig. 1:**
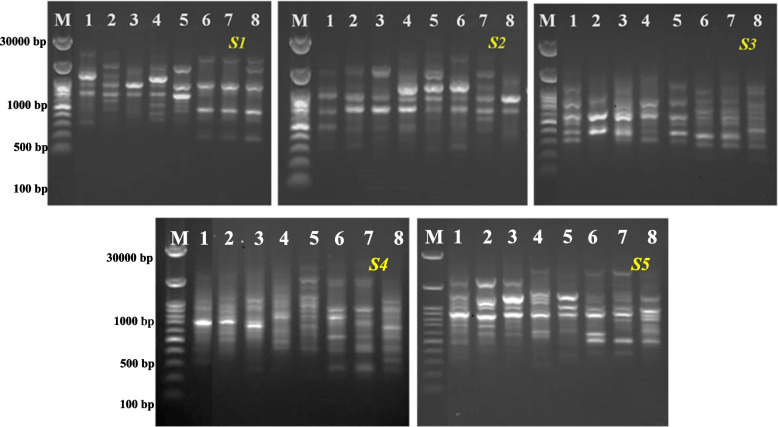
Impact of 5ALA on reproducible DNA fragments of faba bean cultivars using ISSR molecular markers (M=DNA Marker; 1=Giza 843; 2= 5ALA at 1mg/L+ Giza 843; 3= 5ALA at 3mg/L+ Giza 843; 4= 5ALA at 6mg/L+ Giza 843; 5= Nubaria 1; 6= 5ALA at 1mg/L+ Nubaria 1; 7=5ALA at 3mg/L+ Nubaria1.; 8=5ALA at 6mg/L+ Nubaria 1)

Using five ISSR primers, Table (7) and Fig. (1) showed how 5ALA affected the two faba bean cultivars under investigation. Every primer was found to detect a distinct number of bands with a wide range of molecular weights and a high polymorphism ratio. Nonetheless, 112 bands (total bands TB) ranging in molecular weight from 2277.73 to 151.63 bp were identified. Additionally, the total band were distributed between 59 polymorphic bands (PB) with an average 11.8, and 31 unique bands (UB) with an average 6.2 and 22 monomorphic bands (MB) with an average 4.4. The highest levels of polymorphism (PB% was 85.72%) were detected by using IS-02 and the lowest levels of polymorphism (PB% was 77.78%) were detected by using IS-03.

**Table 7:** Impact of 5ALA on reproducible DNA fragments of faba bean cultivars using ISSR molecular markers

Primers	Marker weights (bp)	Amplified bands				PB %
		**TB**	**MB**	**UB**	**PB**	
**(CT)**_**8**_** GC**	**1690.85 – 151.63**	**23**	**5**	**6**	**12**	**78.26%**
**(CA)**_**6**_** AC**	**2277.73 – 192.93**	**21**	**3**	**7**	**11**	**85.72%**
**(GA)**_**6**_** CC**	**1175.07 – 294.12**	**18**	**4**	**4**	**10**	**77.78%**
**(GT)**_**6**_** CC**	**1606.11 – 286.01**	**24**	**5**	**7**	**12**	**79.17%**
**(CAC)**_**6**_** GC**	**1928.47 – 293.72**	**26**	**5**	**7**	**14**	**80.77%**
**Total**		**112**	**22**	**31**	**59**	**-**
**Average**		**22.4**	**4.4**	**6.2**	**11.8**	**80.34%**

**Fig. 2:**
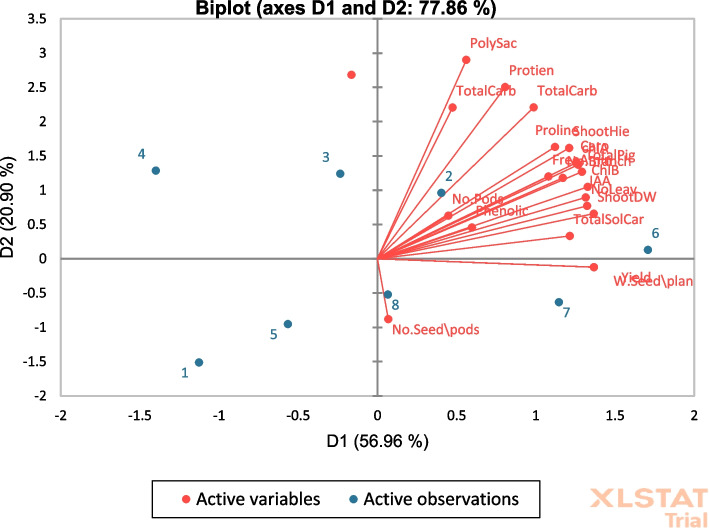
Biplot graph of studied parameters and treatments, showing the first two principal component analysis (PCA) dimensions (D1 and D2)

A principal component analysis (PCA) biplot was performed to represent the impact of the studied applications on the studied variables. The both two PCA diminution 1 (D 1) and diminution 2 (D 2) showed 56.96% and 20.90% of data variability, respectively (Fig. 2). Results shown in Fig. 2 reveal that foliar applications of 5ALA especially at 1mg/L on two faba bean cultivars (as mentioned by two blue dots 2 and 6) caused high variation on the studied parameters. The high variability between the 5ALA treatments indicated the role of 5ALA at 1mg/L in improving the growth parameters and physio-biochemical traits of bean plants. Therefore, 5ALA treatment at 1mg/L played a significant role in improving the growth indices of two faba bean cultivars.

Table 8 shows that the correlation coefficients range from −0.629 to 1, with 1 indicating a perfect positive correlation whereas values closer to −1 indicating a stronger negative correlation. These insights can help in understanding the interdependencies among different plant physiological and biochemical traits. Table 8 shows strong positive correlations, variables such as chlorophyll A (chlA), chlorophyll B (chlB), and carotenoids (Caro) indicating very high positive correlations with each other, and a close relationship in their biosynthesis or regulation pathways. On the other hand, there is a notable negative correlation between the number of seeds per pod and several other variables like the number of branches and shoot height, suggesting that as the number of seeds per pod decreases, other factors tend to increase. The statistical significance correlations marked in bold are statistically significant at an alpha level of 0.05, meaning there is less than a 5% probability that these correlations are due to random chance.

**Table 8:**
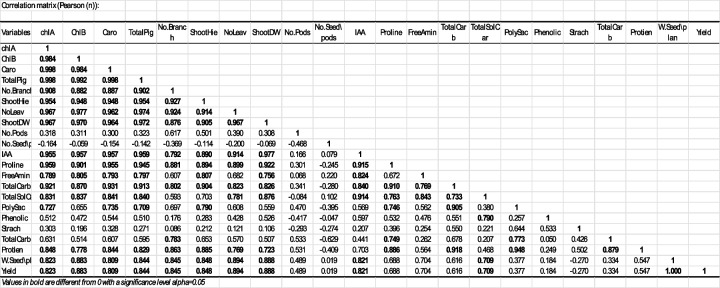
Correlation coefficients among faba bean growth and yield components

## Discussion

### Cultivars difference

Results showed significant difference in most estimated parameters between two faba bean cultivars under investigation. It was noted that Nubaria 1 cv. was characterized by higher significant values in photosynthetic pigments, indole acetic acid, free amino acids, total soluble sugars, seed yield, straw yield, phenolic content and anti-nutritional substance (vicine) relative to Giza 843 cv.The disparities in performance for yield and qualities between the two cultivars may be linked to their genetic background. Recently, Gerema [45] and Essa [46] demonstrated that varieties differ in their growth and development and these changes are due to the plant's morphological, physiological, and biochemical processes. Furthermore, genetic and growing environments (soil conditions, climate factors, and agronomic practices) affect various quality traits, including anti-nutrients of peas and faba bean [47, 48].

### Effect 5ALA on growth, quantity and quality of two cultivars of faba bean

It was noted from collected results that 5ALA at low dose (1mg/l) significantly increased—in most cases- vegetative growth parameters, photosynthetic pigments, some chemical composition of leaf tissues (indole acetic acid, carbohydrate, proline, free amino acid), seed yield and some chemical composition of the yielded seeds (carbohydrate, protein, phenolic content) and decreased vicine content of two cultivars of faba beans under investigation. The discussion of these results based on foliar application of 5ALA at low doses is known to enhance plant growth [18] as a result of improvement of absorption of water and minerals [23] or increasing production of proteins due to boost in nitrogen absorption and assimilation [49]. In addition, 5ALA induced the expressions of enzymes involved in Calvin cycle under water deficit stress thus boosted growth and consequently yield [18]. At the same line, Kosar et al. [50] stated that application of 5ALA boosted wheat grain yield by improving plant growth, chlorophyll a, chlorophyll b, and nitrogen contents of root and leaf at different levels of water regimes. Several reports suggested that exogenous application of 5ALA at low concentrations has stimulatory effects on plant growth that may be ascribed to increases in chlorophyll synthesis, photosynthetic efficiency, net photosynthetic rate, and reduced respiration [3, 51, 52] photosynthetic electron transport activity and Rubisco activity [3, 53]. Besides, priming with 5ALA also preserves plant photosynthesis by suppressing chlorophyll degradation and increasing photosynthetic rate [54, 55]. Moreover, 5ALA acts as a precursor of porphyrin biosynthesis such as chlorophyll [4, 10, 14, 20, 56], and also modulates the porphyrin biosynthesis [6]. In addition, exogenous 5ALA significantly increased carotenoide biosynthesis by up-regulating the levels of gene expression of phytoene synthase 1, phytoene desaturase, geranylgeranyl diphosphatesynthase, and lycopeneb-cyclase [57].

Regarding positive effect of 5ALA on chemical composition of leaf tissues and yielded seeds, previous researches have demonstrated a significant role for 5ALA at low concentrations, as an elicitor in enhancing crop quality and yield through regulating plant cell metabolism even under stress [55, 58, 59]. In addition, earlier researches documented that soaking seeds with 5ALA promotes root elongation of *Arabidopsis thaliana* [9] and *Cajanus cajan L.* seedlings by regulating auxin transport [60]. Application of 5ALA caused accumulation of simple sugars and maintenance of starch content in the leaves of sunflower [14] and rapeseed [11]. It is well confirmed that 5ALA induced the expressions of enzymes involved in Calvin cycle that regulated the synthesis of carbohydrates [18]. It was demonstrated that exogenous application of 5ALA increased production of sugars, amino acids, organic acids, that played an important role in osmotic regulation and protein metabolism [1, 21, 57, 61]. Moreover, exogenous application of 5ALA up-regulateddelta-1-pyrroline-5-carboxylate synthase (P5CS) that controls the rate-limiting step of glutamate-derived proline biosynthesis in oilseed rape (*Brassica napus*) [62]. Likewise, 5ALA is capable of inducing protein content via inducing the activities of nitrate reductase (NR), glutamate synthase (GOGAT), glutamine synthetase (GS), and glutamate dehydrogenase (GDH), and decreasing the activity of nitrite reductase (NiR) [63, 64]. In addition, 5ALA acts as a plant growth regulator which can increase different kinds of non-enzymatic antioxidants such as flavonoids and phenolics in plant tissues [60] in tomato [65], lettuce [66], and purslane [23]. Tavallali et al. [23] demonstrated that 5ALA treatment regulated phenylalanine ammonia-lyase, chalcone synthase, andchalcone isomerase activities and increased concentrations of flavonoids, anthocyanins, and polyphenolics.

With regard to the anti-nutritional compounds known as vicine, Bjerg et al. [67] pointed out that the presence of chemicals responsible for favism in faba bean seeds appeared to be regulated by both genetic and environmental variables. The impact of some treatments on the metabolic pathway of vicine precursor (orotic acid) generation, which is in responsible for forming the pyrmidine ring of these harmful components, may be the cause of the drop in the vicine level [68].

## Conclusion

It could be concluded that 5ALA treatment significantly improved most of the vegetative growth parameters and yield of the two tested faba bean cultivars via stimulating biosynthesis of the plant’s bioactive components like photosynthetic pigments, indole acetic acid, proline and free amino acids. Moreover, 5ALA gave higher seed and straw /fed, total carbohydrate and protein, and phenolic contents accompanied by significant decreases in vicine content of two faba bean cultivars relative to the corresponding controls. However, these increases in the majority of the parameters under investigation are going against the direction of the 5ALA concentration. The lowest concentration of 5ALA (1 mg/L) was the most significant treatment in both cultivars. Regarding anti-nutritional substance called vicine, 5ALA at 3 mg/L caused significant decrease in vicine content of Giza 843 cv. relative to control. Moreover, Nubaria 1 cv. was more effective in responding to 5ALA at 1 mg/L than Giza −843 cv. faba bean plants.

Although 5ALA acted as a regulator of plant growth and increased plant productivity at low concentrations, increasing attention must be paid to effect of 5ALA on the main strategic crops at a lower level than 1 mg/L and to explore the different mechanisms behind 5ALA’s functions.

## Data Availability

Dear Editor. All data supporting the findings of this study are available within the paper.
